# Supplementary material for: Uptake and retention in HIV care among pregnant and postpartum women living with HIV under different eras of vertical transmission prevention policies in sub-Saharan Africa: a systematic review and meta-analysis
Source: medRxiv. 2026 Apr 8:2026.04.02.26350030. Preprint. [Version 1] doi: 10.64898/2026.04.02.26350030 (PMC13086076; doi:10.64898/2026.04.02.26350030)
Supplement: Supplementary file 1 [file NIHPP2026.04.02.26350030v1-supplement-1.pdf]

medRxiv preprint doi: https://doi.org/10.64898/2026.04.02.26350030; this version posted April 8, 2026. The copyright holder for this preprint (which was not certified by peer review) is the author/funder, who has granted medRxiv a license to display the preprint in perpetuity.
It is made available under a CC-BY 4.0 International license .

Supplementary Table

Supplementary Table 1. Retention in HIV care among pregnant women at 6-, 12- and 24 months follow up in a systematic review of studies of prevention of vertical transmission of HIV in sub-Saharan Africa from 2010-2023

.				Percentage retained		
**Author & publication year**	**Country**	**PVT policy**	**N**	**6 months**	**12 months**	**24 months**
Ahoua et al. (2020)[35]	Mozambique	Option B+	31186		60	
Chan et al. (2016)[39]	Malawi	Option B+	914	81		
Chimwaza et al. (2021)[40]	Zimbabwe	Option B+	388	73		
Clouse et al. (2013)[12]	South Africa	Prior to Option B+	273	57		
Dzangare et al. (2016)[41]	Zimbabwe	Option B+	282	70		
Erlwanger et al. (2017)[42]	Zimbabwe	Option B+	1113		39	
Etoori et al. (2018)[43]	Swaziland	Option B+	665	80	71	53
Ford et al. (2017)[44]	Zimbabwe	Option B+	386	85	79	
Haas et al. (2016)[47]	Malawi	Option B+	29768	77	69	61
Hosseinipour et al. (2017)[48]	Malawi	Option B+	447	70		
Joseph et al. (2017)	Zimbabwe	Option B+	547	63	51	
Kim et al. (2015)	Malawi	Prior to Option B+	1129	82		
Llenas-García et al. (2016)[52]	Mozambique	Option B+	243		42	
Lyatuu et al. (2021)[53]	Tanzania	Option B+	15586	82		
Muhumuza et al. (2017)[66]	Uganda	Option B+	2169	74	67	
Schwartz et al. (2015)[59]	South Africa	Option B+	100	62		
Sam-Agudu et al. (2017)[28]	Nigeria	Prior to Option B+	497	44		
Phiri et al. (2017)[63]	Malawi	Option B+	1269		74	56
Phillips et al. (2018)[64]	South Africa	Prior to Option B+	617		90	82
Phillips et al. (2014)[23]	South Africa	Prior to Option B+	358	68		
Myer et al. (2012)[33]	South Africa	Prior to Option B+	490		75	
Myer et al. (2018)[64]	South Africa	Prior to Option B+	471		85	
Humphrey et al. (2022)[69]	Kenya	Option B+	856	16	15	
Teasdale et al. (2022)[70]	Kenya	Option B+	543	91		
Azanaw et al. (2023)[77]	Ethiopia	Option B+	403	99	91	
Bengtson et al. (2024)[91]	Malawi	Option B+	402	77		
Greenberg et al. (2025)[81]	Lesotho	Option B+	304	81	77	40
Mutale et al. (2025)[103]	Malawi	Option B+	49	88		
Njah et al. (2023)[83]	Cameroon	Option B+	560	82	69	
Odongo et al. (2023)[84]	Uganda	Option B+	368		95	

medRxiv preprint doi: https://doi.org/10.64898/2026.04.02.26350030; this version posted April 8, 2026. The copyright holder for this preprint (which was not certified by peer review) is the author/funder, who has granted medRxiv a license to display the preprint in perpetuity.
It is made available under a CC-BY 4.0 International license .

Young et al. (2024)[86]	Nigeria	Option B+	1535	97	94	90

Summary estimates at 6 months - 56.1%, 12 months – 60.0%.
